# Keyword Extraction: A Modern Perspective

**DOI:** 10.1007/s42979-022-01481-7

**Published:** 2022-12-15

**Authors:** Tadashi Nomoto

**Affiliations:** National Institute of Japanese Literature, Tachikawa, Tokyo 190-0014 Japan

**Keywords:** Historical survey, Meta-analysis, Keyword extraction, Automatic indexing, Natural language processing, Information extraction, Text generation

## Abstract

The goal of keyword extraction is to extract from a text, words, or phrases indicative of what it is talking about. In this work, we look at keyword extraction from a number of different perspectives: Statistics, Automatic Term Indexing, Information Retrieval (IR), Natural Language Processing (NLP), and the emerging Neural paradigm. The 1990s have seen some early attempts to tackle the issue primarily based on text statistics [13, 17]. Meanwhile, in IR, efforts were largely led by DARPA’s Topic Detection and Tracking (TDT) project [2]. In this contribution, we discuss how past innovations paved a way for more recent developments, such as LDA, PageRank, and Neural Networks. We walk through the history of keyword extraction over the last 50 years, noting differences and similarities among methods that emerged during the time. We conduct a large meta-analysis of the past literature using datasets from news media, science, and medicine to business and bureaucracy, to draw a general picture of what a successful approach would look like.

## Introduction

The notion of ‘keyword’ has long defied a precise definition. Boyce et al. [7] called it *a surrogate that represents the topic or content of a document,* which in turn gives rise to another question: What is a topic or content? Which is equally elusive. History witnessed the rise of two major schools of thought, one in terminology science (TS) and the other in information retrieval (IR). The two have crisscrossed each other as they progressed in their scientific endeavor. Terminologists are generally concerned with finding terms that are specific to a particular technical domain, useful to organize knowledge relating to that domain, while people in information retrieval are focused more on identifying terms (which they call indexing terms) capable of distinguishing among documents to improve document retrieval.

**Fig. 1:**
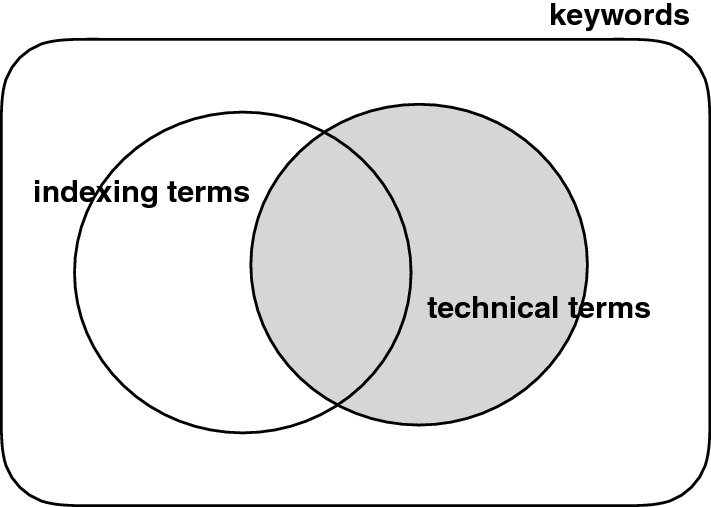
Keywords, technical terms, and indexing terms

Despite some fundamental differences, there is one principle that cuts across TS and IR: that keywords are terms that reside in the document. Hulth [24] reported, however, that people, when asked to provide keywords for their own scientific writings, picked words not only from their work, but also drew upon their own personal knowledge, suggesting that keywords may not be confined to the text alone. In Sect. 4, we show that this is indeed the case, drawing on evidence from data from online sources. We argue that there is more to keywords than indexing and technical terms (Fig. 1).

In this work, we use term *keyword* as an overarching term to refer to linguistic expressions that take on one or more of the following roles.

- **Terminology:** words or phrases that are used in a specific domain to denote a particular technical idea; e.g., *phosphogypsum*, *progressive taxation*, *return on equity*, *Planck constant*, *sarcoma*, *carcinogen*.
- **Topics:** terms and labels that are part of a set of concepts systematically assembled under a particular classification policy; e.g., Wikipedia category names, Dewey Decimal Classification.
- **Index terms:** terms indicating major concepts, ideas, events, and people, referred to in a document or book; e.g., *JFK*, *Martin Luther King, Jr.*, *Malcolm X*.
- **Summary terms:** words or phrases that are meant to serve as a quick description of the content; e.g., *global warming*, *deforestation*, *extreme weather*.

This work is intended as an introduction to major ideas that have evolved and shaped the field for the last 50 years, which retain relevance to this day. Some of them came from TS, others from IR or from computational linguistics. One may ask why we need another survey of keyword extraction, given that there have been a number of efforts out there with an aim similar to ours, in particular Firoozeh et al. [19].^1^ The reason has to do with one problem they all share: the failure to recognize the limits of extractive methods. Keywords arise not only from inside the document but also from outside, i.e., an external source the author has access to. To be able to address keyword extraction requires delivering solutions to both types of keywords. We will see how some of the recent developments such as a generative paradigm based on deep learning, can address the challenge with their unique ability to ‘invent’ keywords as needed.

Another aspect of a keyword, often dismissed as less important in the past literature, including UNESCO [59]—which forms a moral foundation for Firoozeh et al. [19] 2—is the length: we will demonstrate empirically that it plays an important role in defining what it takes to be a keyword, and argue that this recognition of a role the length plays provides a key to solving what Hasan and Ng [23] called ‘conundrums in keyword extraction.’

Our overall contribution lies in putting in a new light aspects of a keyword that have been left largely unexplored and untouched in previous like-minded surveys. In the final section, we will line up major methods that emerged over the past 50 years, and compare them against one another, giving some idea of where they stand in terms of performance and design choices.

## History

### Automatic Term Indexing

Term indexing, described by Boyce et al. [7] as a field of study concerned with finding surrogates that represent the topic or content of documents, remains as relevant as ever in information retrieval today. TFIDF, a widely acclaimed method for finding important words, came into being in the early 1970s when Salton and Yang [51] proposed to measure the importance of a word using the following formula:

1 $$\begin{aligned} I_{ij} = f({\it w}_{ij})\,\log _2 \dfrac{n(D)}{\sum _j g({\it w}_{ij})}. \end{aligned}$$

It marks a huge break from approaches prevalent at the time that were mostly focused on the frequency of terms in and across documents. TFIDF takes the importance of a word in document as consisting of two components. The first component $f({\it w}_{ij})$ is the frequency of a word *i* in the document *j*, also known as TF. The second component $\log _2(\cdot )$, known as the inverse document frequency or IDF, is to indicate how uncommon the word is. $g({\it w}_{ij})$ = 1 if ${\it w}_{ij}$ appears in document *j*; otherwise 0. Therefore, $\sum _j g({\it w}_{ij})$ equals the number of documents containing ${\it w}_i$. *n*(*D*) is the total number of documents in a collection.

The discovery of TFIDF was followed a year later by another formulation [52], which expanded and refurbished the idea to deal with two-term keywords:

2 $$\begin{aligned}&I_{(ik)j} = \dfrac{f({\it w}_{ij})\, f({\it w}_{kj})}{2}\nonumber \\&\quad \biggl \{ \log _2 n(D) - \dfrac{log_2 \sum _j g({\it w}_{ij}) + \log _2 \sum _j g({\it w}_{kj})}{2} \biggr \}. \end{aligned}$$

Note that this can be transformed into

3 $$\begin{aligned} I_{(ik)j} = \dfrac{1}{2} \biggl \{ f({\it w}_{ij})\, f({\it w}_{kj}) \biggl ( \log _2 \dfrac{n(D)}{\sum _j g({\it w}_{ij})} + \log _2 \dfrac{n(D)}{\sum _j g({\it w}_{kj})} \biggl )\biggl \}, \end{aligned}$$

where we are able to see the TF and IDF components more clearly. $I_{(ik)j}$ denotes the importance of a two-word term ${\it w}_i\,{\it w}_k$ in document *j*.

Meanwhile, Robertson and Jones [48] defined the term importance in terms of how well it served the document search. The importance of term ${\it w}_{ij}$ was given by

4 $$\begin{aligned} I_{ij} = \log \dfrac{\Bigl ( \dfrac{r}{R-r} \Bigr )}{\Bigl ( \dfrac{n-r}{N - n - R + r}\Bigr )}, \end{aligned}$$

where for a given query *q*

$$\begin{aligned} \begin{aligned} N&=\text {the number of documents,}\\ R&=\hbox { the number of relevant documents for}\ q\text{,}\\ n&=\hbox { the number of documents containing term}\ {\it w}_{ij}\text{,}\\ r&=\hbox { the number of relevant documents with}\ {\it w}_{ij}.\\ \end{aligned} \end{aligned}$$

The problem with the approach, from a standpoint of keyword extraction, is that to determine the term importance, one needs to find relevant documents for query *q*, which can be challenging, because this would require asking humans to make a judgement on relevance for each of the documents collected. Another issue is that a term will no longer have a unique score as it is made relative to a query: the use of a different query may result in a different score even for the same term. This is troublesome, as it implies that the importance of a word cannot be determined without a reference to a query. These issues inherent to the idea make it unlikely that a relevance-based term indexing would meaningfully contribute to keyword extraction.

Salton et al. [52] takes somewhat a different route, exploring what they call the discrimination value analysis. The idea is based on the intuition that one can determine the importance of a term by looking at how well it is able to discriminate documents in a collection: a good indexing term is one that would separate documents from one another, making a collection sparse.

Assume that we have a document represented as a vector which keeps track of the frequency of every term we find in the document. Averaging the in-document frequency of each term will give us a centroid vector, $C=(c_1,c_2,c_3 \cdots c_i \cdots c_n )$, where each element $c_i$ looks like

5 $$\begin{aligned} c_i = \dfrac{1}{n} \sum _{j=1} f({\it w}_{ij}), \end{aligned}$$

*n* is a number representing how many unique terms we have in the collection, and *f*(*w*) the in-document frequency of *w*. Define the density of a collection by

6 $$\begin{aligned} Q=\sum _{i=1}^M \cos (C,D_i), \end{aligned}$$

where $\cos (\cdot ,\cdot )$ denotes the cosine similarity, *C* a centroid, and $D_i$ a document vector, where

7 $$\begin{aligned} D_i = \bigl ( f({\it w}_{1i}), f({\it w}_{2i}), f({\it w}_{3i}), \ldots , f({\it w}_{ni}) \bigl ). \end{aligned}$$

*M* indicates the number of documents the collection contains. Define a function $DV_k$ for ${\it w}_k$ as

8 $$\begin{aligned} DV_k = Q_k - Q. \end{aligned}$$

For a given term ${\it w}_k$, $Q_k$ is a *Q* score one gets by setting $f({\it w}_{ki} )=0$ for every $D_i$. $DV_k > 0$ means that *w* has the ability to discriminate documents (because its removal from the collection causes an increase in density, making documents more similar). Salton et al. [52] define the discriminative value of term ${\it w}_k$ in document *j* by

9 $$\begin{aligned} I_{kj} = f({\it w}_{kj}) \cdot DV_k. \end{aligned}$$

The authors reported that their approach, when applied to three datasets, CRANFIELD [47], MEDLARS [16], and TIME [52], led to an improvement by 10% over an approach which only makes use of the term frequency.

Nagao et al. [41], inspired by $\chi ^2$ statistic, came up with an interesting alternative

10 $$\begin{aligned} X^2 = \sum _j \dfrac{f({\it w}_{ij}) - m_{ij}}{m_{ij}}, \end{aligned}$$

where

11 $$\begin{aligned} m_{ij} = \dfrac{\sum _j f({\it w}_{ij})}{\sum _{ij} f({\it w}_{ij})} \sum _i f({\it w}_{ij}). \end{aligned}$$

$X_i^2$ represents the importance of term *i*. The idea is that if its frequency $f({\it w}_{ij})$ deviates from its expected frequency $m_{ij}$, we take it as worthy. One caveat is that one must have a large collection of documents, to guarantee that an estimated $X^2$ follows $\chi ^2$ the distribution.

The idea was further explored by Matsuo and Ishizuka [36], who proposed to replace Eq. 10 with

12 $$\begin{aligned} \chi ^2 ({\it w}) = \sum _{g\in G} \dfrac{(f^\prime ({\it w},g) - f({\it w}) p(\cdot , g))^2}{f({\it w})p(\cdot , g)}. \end{aligned}$$

$f^\prime ({\it w},g)$ indicates how many sentences there are that contain *w* and *g* together. *f*(*w*) is the count of sentences in a document which contains *w* and $p(\cdot ,g)$ the probability that any given term appears together with *g* (in a sentence), i.e., $\sum _i^V p({\it w}_i, g)$, with *V* indicating the total number of uniques words in a collection. $f({\it w})p(\cdot ,g)$ thus corresponds to the expected frequency of *w* co-occurring with *g*. *G* is a pre-defined set of frequent terms in a document (with stopwords and other minor words removed). The authors’ goal was to find how far the observed co-occurrence frequency of *w* and *g* deviates from its expected frequency. The further it veers off, the greater its significance. Matsuo and Ishizuka [36] went on to suggest using the following in place of Eq. 12:

13 $$\begin{aligned} \chi ^\prime ({\it w}) = \chi ^2 ({\it w}) - \max _{g\in G} \dfrac{(f^\prime ({\it w},g) - f({\it w})p(\cdot ,g))^2}{f({\it w})p(\cdot ,g)}. \end{aligned}$$

The formula penalizes a term if its chi-squared value is backed by only a small number of high frequency terms. Thus, if we have two terms A and B ($\in G$), and if A occurs only with B and not any other member of *G*, $\chi ^2 (\textsf {A})$ will get a high score, but $\chi ^\prime (\textsf {A})$ will get 0.

**Table 1:** Performance of $\chi ^\prime$ vs. baselines

	TF	$\chi ^\prime$	TFIDF	Keygraph
Precision	0.53	0.51	0.55	0.42
Recall	0.48	0.62	0.61	0.44

Table 1 gives some sense of how well it works. The test was done using 20 scientific papers. We observe that $\chi ^\prime$ is doing almost as good as TFIDF, without relying on the document frequency, which the latter requires. Every material that $\chi ^\prime$ makes use of comes from inside the document. The table also shows the performance of keygraph, another method based on a co-occurrence metric, described below. TF is the simplest of all, relying only on the term frequency.

Ohsawa et al. [43] are the earliest attempt (to our knowledge) to leverage the notion of *word graph* to extract keywords, an approach they termed ‘*keygraph*.’ A word graph is an interconnected network of words built by linking words based on how closely a pair of words are associated, e.g., the number of times the pair co-occurs in a sentence. Ohsawa et al. [43] defined the strength of association $\mathscr {A}$ between a pair of words, ${\it w}_1$ and ${\it w}_2$, using the formula

14 $$\begin{aligned} {\mathscr {A}}({\it w}_i,{\it w}_j) = \sum _{s\in D} \min (|{\it w}_i |_s, |{\it w}_j|_s). \end{aligned}$$

*D* is a set of sentences in the document, $|{\it w}_i|_s$ represents the number of times ${\it w}_i$ occurs in sentence *s*, and similarly for $|{\it w}_j|_s$. $\min (x,y) = x \text { if } x \le y;\; \text  { otherwise }y$. $\mathscr {A}$ was meant to ignore weakly connected pairs.

**Fig. 2:**
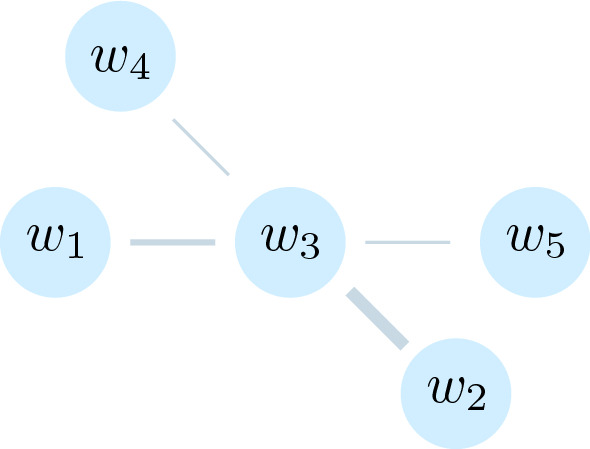
A word graph

**Fig. 3:**
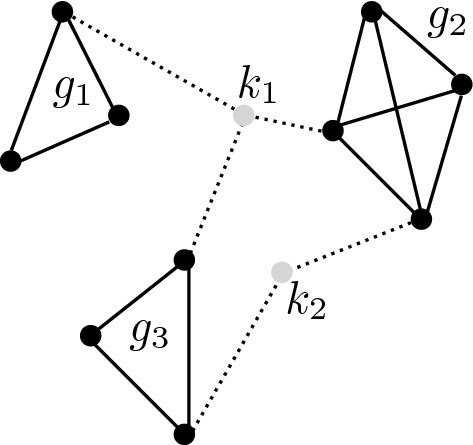
Subgraphs

Figure 2 shows a word graph consisting of five nodes, each corresponding to a word, and a link between nodes, indicating that corresponding words occur together in some of the sentences in *D*. The width of a link indicates the strength of association as given by Eq. 14.

The approach further divides a word graph into a set of subgraphs which the authors claimed to correspond to distinct topics the writer may have had in mind when penning the document. Figure 3 gives some idea of what they are like. Each node (or vertex) represents a word, with an edge (solid line) signaling the presence of a co-occurrence relation between words, indicating that there are sentences in which they appear together. A subgraph is a set of nodes (words) where every member of the set is linked to every other. Ohsawa et al. [43] assume that a keyword is a word that participates in multiple subgraphs, such as words denoted by  $k_1$ and $k_2$ in Fig. 3. The following is a formal definition of the importance of word *w* that encapsulates the idea:

15 $$\begin{aligned} I({\it w}) = 1 - \prod _{g\in G} \Biggl ( 1 - \dfrac{B({\it w},g)}{N(g)} \Biggl ), \end{aligned}$$

where

$$\begin{aligned}&B({\it w},g)= \sum _{s\in D} |{\it w} |_s \cdot |g - {\it w} |_s, \\&N(g) = \sum _{s\in D} \sum _{{\it w}\in s} |{\it w} |_s \cdot |g - {\it w} |_s, \end{aligned}$$

and

16 $$\begin{aligned} |g - {\it w} |_s = {\left\{ \begin{array}{ll} |g |_s - |{\it w} |_s &{} \text {if } {\it w} \in g\\ |g |_s &{}\text {otherwise}.\\ \end{array}\right. } \end{aligned}$$

*g* stands for a subgraph in document *D*. $G=\{g_1, g_2, \dots , g_m\}$. Note that

$$\begin{aligned} |g|_s = \vert \{{\it w} \mid {\it w} \in g \} \cap \{{\it w} \mid {\it w} \in s \}|. \end{aligned}$$

*B*(*w*, *g*) indicates how many times *w* co-occurred with a member of a subgraph *g*. *N*(*g*) is a normalizing factor. Intuitively, *I*(*w*) says that the greater the number of subgraphs is that contain *w*, the more important it will be, a proposition which could be interpreted as saying that important words are those whose occurrence is widespread across the document. According to Ohsawa et al. [43], the approach performed on par with TFIDF in document retrieval.

We conclude the section by pointing out that the past approaches to term indexing share a particular view about its nature [27]: an indexing term is something that resides in a document, occurs frequently across documents, and exhibits a distinct distributional pattern. Term weighting schemes proposed in the Automatic Indexing literature all reflect this principle one way or another.

### Computational Linguistics

**Table 2:** Frequencies of terminological terms (Table 1 in Justeson and Katz [26])

	Term length (in words)			
domain	1-gram	2-gram	3-gram	4-gram
fiber optics	43	109	36	12
medicine	88	80	22	10
physics & math	41	125	29	5
psychology	64	120	12	4

Justeson and Katz [26] were the first attempt to look into linguistic properties of technical terms. They examined the terminology used in technical dictionaries from various domains, including fiber optics, physics and mathematics, medicine, and psychology. The study concluded that noun phrases accounted for 92.5–99% of the technical terms found, with about 70% of them having more than one word; there were a few cases where they accompanied adjectives and to a lesser degree, prepositions; but there was no instance which involved verbs. Table 2 summaries their findings. Most of the terms are made up of two words with an exception of medical terms (for which the authors attempted a linguistic explanation).

Not surprisingly, the authors were more into developing linguistics of keywords than engineering a solution, as is manifest in questions they asked, such as ‘Why do technical terms resist the use of conjunctions, prepositions, and adverbs?’ Their answer to that was that technical terms take shape under two opposing linguistic forces, one that pushes them to become shorter and the other pulling them towards more transparency. Because none of the excluded types (terms which include verbs, conjuncts, and prepositions) are able to accommodate the demand of either of the two forces, they are disfavored.

The authors further suggested the following two tests to identify technical terms: (1) whether a term is two word long; and (2) whether it matches a regular expression of the form:

17 $$\begin{aligned} ((A|N)+ | ((A | N)NP)?)(A | N)*)N, \end{aligned}$$

‘*A*’ denotes an adjective, ‘*P*’ a preposition, and ‘*N*’ a noun. For details on linguistics notions, refer to Manning and Schütze [31].

Daille et al. [15] generally echoed what was found in Justeson and Katz [26], though they argued that technical terms were something built out of basic multi-word units (MWU) via compositional operations, and went on to say that a complex multi-word term such as *geostationary communication satellite* was the result of combining two MWUs, *geostationary satellite* and *communication satellite.* Salton [53] took a step further, suggesting that we should include discontiguous terms, i.e., those made up of elements separated by some intervening words, such as ‘*building dictionary*’ from ‘*a building of a dictionary.*’

**Table 3:** Potential glossary items (Table 1 in Park et al. [45])

syntactic Pattern	Example
AN	Genuine part
NN	Sport utilities
AAN	Heavy commercial use
ANN	Rear wiper blade
NNN	Emission control system
AANN	Other qualified service technician
ACAN	Unpaved or dusty roads
ANNN	Automatic transmission fluid level
NNNN	Engine oil fluid level
AANNN	New personalized oil reset percentage
AACAN	Certain frontal or near-frontal collision
ACAAN	Ambient and wide open trouble
NNNNN	Steering wheel fan speed control

Park et al. [45] took on the issue from a somewhat different angle. Their primary interest was in glossary extraction, where a main goal was to locate and extract terms related to a specific domain. What made their work different was a set of syntactic patterns they used to identify candidate terms, examples of which are shown in Table 3. Of a particular interest is the use of conjuncts (i.e., *and, or* in ACAN, AACAN and ACAAN)  which Justeson and Katz [26] explicitly argued against. The motivation for using a particular set of syntactic patterns primarily came from their need to work for a specific domain. To improve a sensitivity to the domain, the authors further proposed a scoring function that favored those of high relevance to a specific domain.

Another interesting idea came from Barker and Cornacchia [4], who promoted a notion of ‘head-driven keyword extraction.’ The idea was to define keywords as NPs (noun phrases) containing most frequent heads. The authors reported a modest improvement over baselines. The work deserves mention because of their unique effort to relate a syntactic theory (then current) to keyword extraction.^3^

## New Perspectives

### PageRank Inspired Approaches

Mihalcea and Tarau [40], on the heels of the success of PageRank, set off on a project they called TextRank. They were interested in finding a way to exploit PageRank in their effort to find keywords in the text. In their adaptation of PageRank, a text is broken into a set of nodes, and edges, with nodes representing words and edges connections among them. The importance of a word is given by the following formula:

18 $$\begin{aligned} S(i) = (1-d) + \sum _{j\in A(i)} \dfrac{{\it w}(j,i)}{\sum _{k\in A(j)} {\it w}(j,k)} S(j). \end{aligned}$$

*i*, *j*, *k* are all words. *A*(*i*) represents a set of words that appear in the proximity of *i*. *w*(*j*, *i*) represents the strength of the bond between *j* and *i* based on their co-occurrence. $d\;$ is what is known as a damping factor.

Imagine that you are at word *i*, thinking about whether to jump to somewhere else in the text. The equation describes the probability of moving to some other word, which is given as the sum of the probability of jumping to some random word and that of moving to some popular word. Intuitively, TextRank reflects an idea that a word you are looking at is important if you see important words around it. TextRank could also be viewed as a modern-day reincarnation of graph-based approaches discussed earlier [36, 43]. Recall that they defined the importance of a word by how often it co-occurs with surrounding words. The only difference is that TextRank takes into account weights of contextual (surrounding) words, which the latter do not.

There is, however, one area where TextRank completely breaks ranks with the conventional widsom. Kageura and Umino [27] argued that the frequency of a term is an important component of an index term. The past work in Automatic Term Indexing tends to agree that a word that occurs frequently often works as an index term. The fact that TextRank has no way of accessing the word frequency implies that words TextRank favors do not necessarily coincide with those that the traditional indexing would find important.

**Table 4:** Details of the corpora (Table 1 in Hasan and Ng [23])

	datasets			
	duc	inspect	nus	icsi
Type news	News	Technical abstracts	Technical papers	Meeting transcripts
# Documents	308	500	211	161
# Words document	876	134	8291	1611
Avg. len. keywords	2.1	2.3	2.1	1.3

**Table 5:** Performance of TextRank and its variants (Table 2 in Hasan and Ng [23])

	F1			
	duc	inspect	nus	icsi
TextRank	9.7	33.0	3.2	2.7
SingleRank	25.6	35.3	3.8	4.4
ExpandRank	26.9	35.3	3.8	4.3
TFIDF	27.0	36.3	6.6	12.1

Hasan and Ng [23] conducted a series of experiments in an effort to find whether TextRank, along with other like-minded approaches such as SingleRank and ExpandRank [62],^4^ has any advantage over TFIDF.^5^ (See Table 4 for some details on the datasets they used.^6^)

Table 5 shows results. What is striking is that graph-based approaches failed to perform at a level comparable to TFIDF, a finding which took Hasan and Ng [23] by surprise. However, we view it as an inevitable consequence of not paying attention to the term frequency and in particular the length of a candidate phrase (we demonstrate that this is the case later in the paper). In this light, Matsuo and Ishizuka [36] and Ohsawa et al. [43], both graph-based, may have worked better if Hasan and Ng had tried them,  as they have a means to access frequency information.

### Using External Knowledge

Using external information has been one of the popular topics in keyword extraction. MAUI [37] is a keyword extractor which has an option to produce keywords from a custom vocabulary. It does this by replacing ngrams with matching descriptors in the vocabulary. One may view the process as a term normalization via external knowledge. Medelyan [37] reported that MAUI, when tested on three datasets, each with a different vocabulary, was able to recover about 40 to 80% of human assigned keywords. The work went on to explore the use of Wikipedia as an external source, which eventually evolved into an approach that treats a Wikipedia title as a keyword. The author suggests the following formula to find a title that matches a given word:

19 $$\begin{aligned} S({\it w},T) = P(T\mid {\it w}) \dfrac{\sum _{c\in C} R(T,c)}{|C |}. \end{aligned}$$

*w* represents a term we want to project into Wikipedia. *C* represents a *context* of *w*, a set of ngrams that co-occur with *w* in a given page. *T* denotes a Wikipedia title. $P(T\mid {\it w})$ is the probability of seeing *T* given *w*. *R*(*x*, *y*) measures how closely *x* and *y* are related. The greater the value, the closer the association between *x* and *y*. The formula looks like the following:

20 $$\begin{aligned} R(x,y) = 1 - \dfrac{\log (\max (|X |, |Y |)) - \log (|X \cap Y |)}{\log (N) - \log (\min (|X |, |Y |))}. \end{aligned}$$

*x* and *y* are ngrams, *X* (or *Y*) a set of incoming links to a page to which *x* (or *y*) is mapped, and $|X |$ its size. *N* is the total number of articles in Wikipedia. Clearly, $R(x,y)=R(y,x)$. We may interpret Eq. 19 as saying: if you have an ngram *w* which relates to multiple Wikipedia pages, pick one which is contextually relevant to *w* and moreover which occurs frequently with *w*. For example, *apple* could mean a number of things; an edible fruit, a place, an American computer company. Equation 19 is intended to disambiguate a term based on a context in which it occurs and on how frequently each of the associated senses is used. Thus, if it is found with words like *orange*, *banana*, *juice*, and *mango*, it is more likely to be mapped into a page representing an apple as an edible fruit.^7^

It is worth noting that MAUI draws upon a technique known as wikification [39]. Wikification is yet another keyword extraction method, which leverages Wikipedia to identify potential keywords. To test if a given word is a keyword, it goes to Wikipedia to see if it is used as an anchor. If it is, then the word will be stored in a set of candidates before they are scored according to a metric it calls *keywordness,* a measure indicating how likely a particular word occurs as an anchor in Wikipedia. The more frequently a word appears as an anchor, the higher it is ranked as a keyword. One drawback is that a newly minted word or a word that entered the public conversation recently is likely to be undervalued, because it has little presence in Wikipedia. This means that to avoid a failure, MAUI needs to keep ‘knowledge-lean’ methods like $\chi ^2$, or TFIDF as a backup.^8^

### Classificatory Keyword Extraction

Classificatory keyword extraction (CKE) represents a class of approaches that work by scanning contiguous spans of a text for keyword, where we visit each word, determining whether or not to include it in a pool of potential keywords. Much of the past and present work, supervised or unsupervised, falls under this category.

In their short paper published in 2018 for AAAI, Florescu and Jin [20] introduced an approach based on a random walk. Like Ohsawa et al. [43], Matsuo and Ishizuka [36], and Mihalcea and Tarau [40], it treated a text as a network of words, with the strength of an association represented by how often they occur together. A major difference between Florescu and Jin [20] and what preceded them lies in their use of latent representations acquired from random walks [46] to determine the strength of connections. The authors reported an improvement over past methods that relied on a word-based representation.

Zhang et al. [1] conceived an approach which combined CRF (Conditional Random Fields) [28] with deep learning machinery (CRF/DL, hereafter). They optimized the model along dual targets. One, given in an IOB format (‘inside-outside-begin’), specifies where a keyword begins and ends. The other indicates whether a particular token is part of a keyword, in a binary format. The idea resulted in a performance better than R-CRF [64], a more faithful implementation of CRF in RNN (Recurrent Neural Networks).^9^ It was unfortunate that Zhang et al. [1], despite their focus on the tweet domain, did not consider problems particular to tweets, e.g., (in-group) abbreviations, slang words, and misspellings, in contrast to Marujo et al. [35], who put these issues at the forefront.^10^

**Fig. 4:**
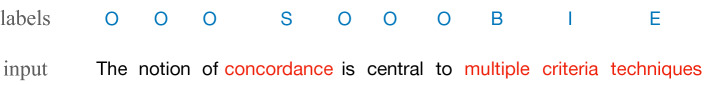
Classificatory DL Extractor (CDL) maps each token in the input into a pre-defined label space, for instance one with $\{O, S, B, I, E\}$, where ‘*S*’ indicates a single-token keyword, ‘*B*’ a beginning of a multi-part keyword (MPK), ‘*I*’ an in-between element and ‘*E*’ an end of an MPK. CDL builds a model in a way that maximizes a quantity $S = \sum _i \log p(\mathbf { y_i} \mid \mathbf {x_i})$, where $\mathbf {y_i}$ denotes $t^+, \text {with } t \in \{{O, S, B, I, E }\}$, $\mathbf {x_i}$ a natural language text. CDL typically makes use of an encoder/decoder architecture of the sort shown in Fig. 5

**Fig. 5:**
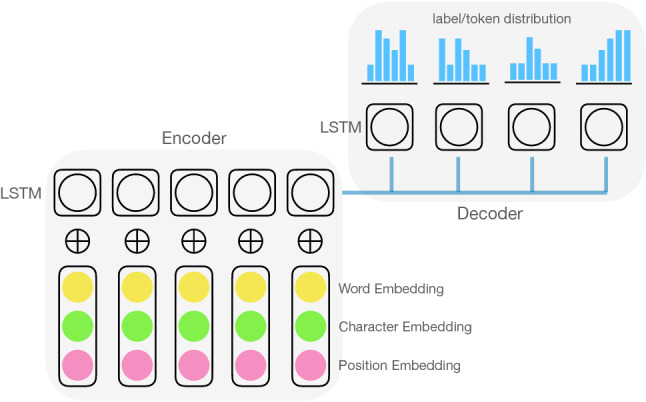
A schematic view of an encoder/decoder neural (sequence to sequence) model. An encoder rolls out a sequence of a recurrent neural network (LSTM), each feeding on a composite representation of a token, and sends the result to a decoder which converts it to probability distributions of labels/tokens, with the output built from labels/tokens with highest probabilities

The idea of Wang et al. [60] centered around how to transfer a keyword model over to a domain for which there is no ground truth available. The authors pursued an extractive approach guided by what is generally known as ‘Generative Adversarial Networks’ or Jensen–Shannon GANs [21]. The idea was to move latent representations of data in an unannotated domain as close to those acquired from a domain for which we know the ground truth, as possible. The model was set up in a way reminiscent of the unsupervised multilingual translation, where multiple independent networks work together to achieve diverse objectives (reconstruction loss, IOB loss, discriminator loss, and the like). The work reported a substantial gain over strong baselines, which included a model similar to CRF/DL [33, 50, 68]. (Figs. 4 and 5 give a high-level picture of how it works.)

### Generative Keyword Extraction

While the external knowledge allows us to move beyond the confine of document, another possibility emerged recently thanks to advances in deep learning (DL), where the focus is more on *generating* keywords. If successful, it may deliver a one-shot solution to acquiring out-of-document and in-document keywords, an issue that plagued the past research. This section introduces a line of work that embraced this particular strategy [12, 38, 67], while giving a cursory look at other DL-based approaches that are essentially extractive in nature [1, 60].^11^

We start with Meng et al. [38]. Assume that we have two RNNs (recurrent neural networks): one encodes the source text (Encoder) and the other generates keywords (Decoder). The input is transformed into hidden representations through the Encoder, which the Decoder takes over to construct an output. While decoding the output, a beam search is typically applied to select candidate keywords. An innovation that Meng et al. [38] bring to the table is a particular objective (loss function) the authors proposed to train the network, namely

21 $$\begin{aligned} {\mathscr {L}} = - \log \biggl ( p_s (y_t \mid y_1 \dots y_{t-1}, x) + p_c (y_t \mid y_1 \dots y_{t-1}, x) \biggl ). \end{aligned}$$

*x* is an input text. $p_s (y_t \mid \cdots )$ denotes the probability that a token $y_t$ is generated using the general vocabulary and $p_c (y_t \mid \cdots )$ the probability that $y_t$ is generated using the vocabulary from the input text. ${\mathscr {L}}$ adds a functionality to the network to be able to reuse part of the input as it creates a keyword. This feature is critical for keyword extraction, because without it, it would be impossible to extract elements from the source. Equally important, it provides the model with the capability to build out-of-document keywords (via $p_s$). It implicitly replicates what MAUI achieved through Wikipedia and a set of mapping rules. Yuan et al. [67] extended Meng et al. [38] by adding a capability to output multiple keywords simultaneously.^12^ In addition, they introduced a learnable switch which allowed them to decide whether to use $p_s$ or $p_c$ during the generation. By contrast, Meng et al. [38] had no control over which one to emit as they relied on the combined probability, $p_s + p_c$.

**Fig. 6:**
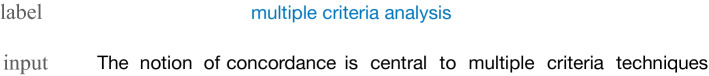
Generative DL Extractor (GDL) takes as input an entire sequence of tokens and generates a keyword, using a training vocabulary, which may or may not appear in the source sequence. The learning proceeds in a way similar to CDL, with an aim to maximize $S = \sum _i \log p(\mathbf { y_i} \mid \mathbf {x_i})$, where $\mathbf {y_i}$ denotes ${w}^+$ (${w} \in \mathbb{V}$), $\mathbf {x}_{\mathbf {i}}$ a natural language text. $\mathbb{V}$ represents a vocabulary (a set of words) derived from training data. Note $\mathbf {x}_{\mathbf {i}} \in \mathbb{V}^{+}$. GDL typically uses the same architecture as CDL (see Fig. 5)

Chen et al. [12] share with Yuan et al. [67] a goal of generating multiple keywords in one fell sweep, but depart from the latter in their emphasis on the diversity, which the former realized using what they called a coverage mechanism (CM), an idea originally from machine translation [56]. CM works as a sort of a ledger to keep a record of how much attention was given to tokens during the encoding. The authors reported that it had successfully prevented a repetitive generation of tokens. Yuan et al. [67], pursuing a somewhat different line while aiming for the same objective, proposed a loss function called orthogonal regularization (OR) [6]

22 $$\begin{aligned} {\mathscr {L}}_\text {OR} = \big \Vert H^\top H\big \Vert _{F}^2.\end{aligned}$$

$H = \langle h_1^d, \dots , h_n^d \rangle$, where $h_i$ is a hidden representation (a *d*-dimensional vector) used to derive the *i*-th keyword. $|\vert \cdot ||_{F}$ is a squared Frobenius norm. *n* is the number of keywords. Minimizing ${\mathscr {L}}_\text {OR}$ has the effect of increasing the diversity among $h_1^d, \dots , h_n^d$, resulting in keywords that vary in form and meaning.

Being able to generate keywords on the fly is a double-edged sword: while it allows you to ‘concoct’ a new word, it may get you inadvertently assigning keywords that are not remotely relevant to what a text is about (for instance, one might end up with a keyword like ‘bible concordance’ from the input given in Fig. 6), a problem that rarely affects the classificatory regime.

**Fig. 7:**
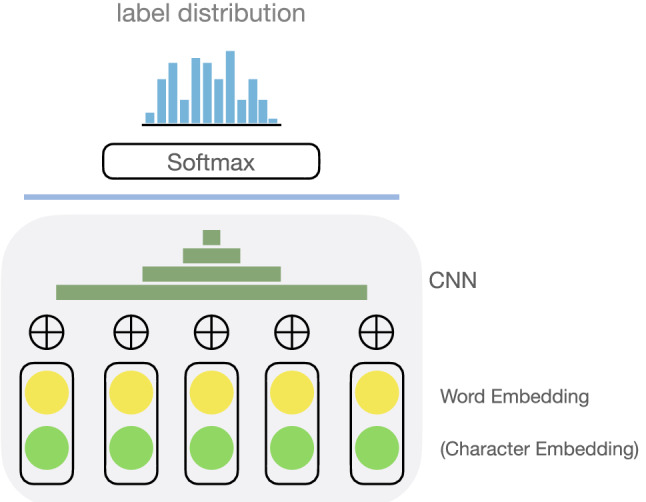
A typical setup to use DL as a text classifier. We project the output of an embedding layer into a softmax layer via a convolutional neural network (CNN), and get a probability distribution of potential categories. The input will be labeled with one or more terms with the highest probability

**Fig. 8:**
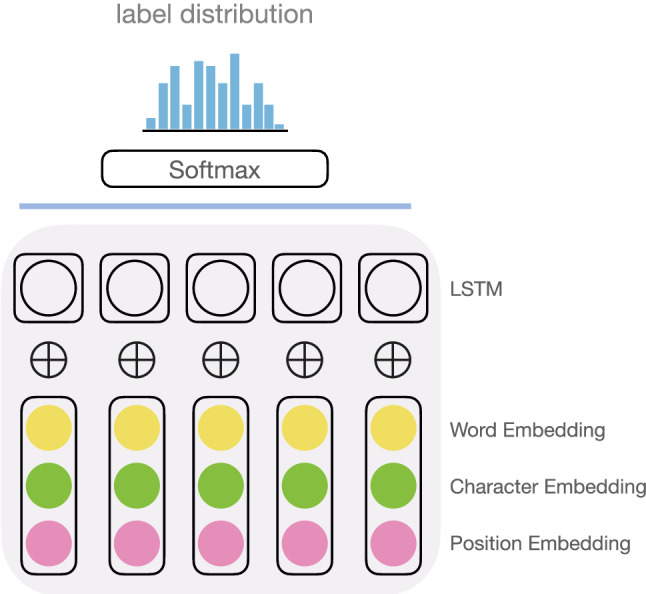
An alternative design (of a kind pursued by Lee et al. [29])

### Keyword Extraction as Text Classification

Text classification (TC) provides another interesting angle from which to look at keyword extraction. One uses TC mostly to associate a document with labels from some pre-defined vocabulary. TC has a long history of research going back many decades, with much of the current effort happening within the realm of DL [14, 29, 61]. While TC is confined to a fixed set of topics, we can turn it into a keyword extractor by enlarging the vocabulary it covers.

A most typical setup to use DL for TC is shown in Fig. 7 [14, 61]. We start with some word embeddings, possibly along with character-level embeddings. We work through convolutional layers (which could be many), and arrive at a condensed representation, which we use to label the text (via a softmax layer). Lee et al. [29] pursued an alternative strategy which made use of LSTM, a recurrent neural network (RNN), in place of CNN, allowing them to incorporate temporal information (Fig. 8). Yin et al. [65], wondering about which approach works better, conducted experiments on various classification tasks ranging from sentiment to relation to entailment classification. They found no significant differences in performance between RNN and CNN.

Yet, some people expressed a concern over applying TC to keyword extraction, worried that the number of categories it had been tested on in the literature was very small (somewhere around 4–10 [14]). There is no work so far, to our knowledge, that addressed the concern. Its ultimate success may hinge on whether it can be extended to work for a large number of categories.

### Working with Textual Cues

An observation that a keyword rarely contains a stop word led Rose et al. [49] to a development of a widely used method known as RAKE.^13^ It extracts keywords by dividing a text into a set of contagious word sequences by stop words, and choosing those that occur most often. Consider a sentence *‘a volcanic explosion killed at least 8 people.’* Assume that one has a list of stop words *‘an’*, *‘killed’*, *‘at’*, *‘least’*, *‘8’*, and *‘.’*. Delimiting the sentence with them gives us

23 $$\begin{aligned}&\parallel \text { an } \parallel \parallel \text { volcanic explosion } \parallel \parallel \text { killed }\parallel \parallel \text { at } \parallel \nonumber \\&\quad \parallel \text { least } \parallel \parallel 8 \parallel \parallel \text { people } \parallel \parallel . \parallel , \end{aligned}$$

and by eliminating the separators, we get24arriving at terms *‘volcanic explosion’* and *‘people.’* While surprisingly simple, it was found to rival more sophisticated approaches like TextRank and Hulth [24].

In a similar vein, KPM or KP-miner [18]^13^ takes anything that occurs between stop words and punctuations as a keyword candidate. A decision on which one to choose is made based on where it occurs in the text and how often it appears. Anything that appears beyond the initial 850-word block of the document and/or those that occur less than three times are discarded. The importance of a candidate is measured by a version of TFIDF, which takes into account additional textual features such as location, and the within-document frequency of candidates.

YAKE [10, 11]^13^ is an outlier among unsupervised systems (which include RAKE and KPM) due to its treatment of the context. The approach is motivated by an intuition that any word that appears in company of many different words is essentially a function word, and thus should be discarded. The claim is an interesting one, because it attempts to identify keywords not by how important they are, but by how *insignificant* they are. They proposed the following to measure the insignificance of a word:

25 $$\begin{aligned} S(x) = \dfrac{R(x) P(x)}{C(x)+\frac{F(x)}{R(x)} + \frac{D(x)}{R(x)}}, \end{aligned}$$

where *x* is a word. *R*(*x*) indicates how many unique tokens are found in company with *x*, *P*(*x*) the position of *x*’s first occurrence. *C*(*x*) records how many times *x* occurs with its initial letter capitalized or appears as an acronym. *F*(*x*) represents the frequency of *x* and *D*(*x*) the number of sentences in which *x* appeared. The lower the value of *S*(*x*), the better. The insignificance of a keyword K (which may involve more than one word) is given as

26 $$\begin{aligned} S(K) = \prod _{x\in K} S(x), \end{aligned}$$

where $$K=x_1 x_2 \cdots x_n$$.

### Going Bayesian: LDA

LDA or Latent Dirichlet Allocation [5]^14^ is another favorite method people turn to. LDA builds a language model, operating on the premise that there is an implicit set of topics that dictate the distribution of words we observe in a document. In LDA, a topic is not a single summary term that describes some aspect of the document, but rather something that represents a probability distribution that spans the entire vocabulary. Words (normally, uni-grams) that occur frequently with a topic are given higher probabilities. In LDA, a topic takes a form like: $z_1$ = {opera, lyrics, hip-hop, jazz, ambient, ...} or $z_2$ = {market, fed, slump, recession, exchange, ...}, each spanning the entire vocabulary, with associated probabilities (suppressed here). You may interpret them as you please. How to make sense of $z_1$ or $z_2$ is entirely left to the user.

In LDA, every word in the document is assigned to some topic:

After a long$_{z8}$ tiring$_{z_{56}}$ week$_{z_{83}}$, House$_{z_{43}}$ Democrats$_{z_{43}}$ decided$_{z_{20}}$ to move$_{z_{12}}$ forward$_{z_{34}}$ with a request$_{z_0}$ for the two articles$_{z_{78}}$ of the impeachment$_{z_{40}}$ against the President$_{z_{43}}$ .

Here, $z_i$ is a topic index. One can have as many or as few topics as he or she wants. There are basically two ways to turn LDA into a keyword extractor. (1) one is to simply take as keywords, words that are most likely to occur under LDA; (2) the other is to select those associated with the most prominent topic. The worthiness of a word under the first approach can be given as

27 $$\begin{aligned} {\mathbb W}^1 ({\it w})= p({\it w}\mid d, \alpha , \beta ) = \sum _{t=1}^{T} p({\it w}\mid t, \beta ) p (t\mid d, \alpha ), \end{aligned}$$

*T* is the number of topics that we assume cover documents. *d* denotes a document. $\alpha$, $\beta$ are parameters responsible for generating probability distributions that determine how likely *w* (word) and *t* (topic  index) occur, or more precisely, $\beta$ represents a matrix of shape $K\times V$ with *K* = the size of topic indices and *K* = the size of the vocabulary.

The second approach can be written as

28 $$\begin{aligned} {\mathbb W}^2 ({\it w}) = {\mathop {\mathrm{max}}\limits _{t \in K}}\, p({\it w}\mid t, \beta ), \end{aligned}$$

where *K* is a set of topic indices $z_i,\dots ,z_k$.

Liu et al. [30] were the first in a line of research [25, 54, 55] working on TopicalPageRank (TPR) to combine PageRank and LDA. TPR takes a form almost identical to Eq. 27

29 $$\begin{aligned} p({\it w}\mid d, \alpha , \beta ) = \sum _{t=1}^{T} R({\it w}\mid t, \beta ) p (t\mid d, \alpha ), \end{aligned}$$

where

30 $$\begin{aligned}&R({\it w}\mid t, \beta ) =\nonumber \\&\quad \underbrace{\lambda \sum _{u\in A(w)} \dfrac{g(u,{\it w})}{\sum _{k\in A(j)} g(u,k)} R(u\mid t, \beta )}_{\textsc {pagerank}} + \underbrace{(1-\lambda )p({\it w}\mid t, \beta )}_{\textsc {lda\, word\, simplex}}. \end{aligned}$$

*g* indicates how strongly words *u* and *w* are associated, *A*(*w*) a set of words that sit in the proximity of *w* (see Sect. 3.1 for further details). The authors reported the composite system performed competitively against LDA and PageRank.

## Where Do They All Stand?: A Meta-Analysis at Scale

In this section, we examine the effectiveness of approaches we discussed above, comparing them side by side on a large number of datasets. We also look at whether performance is affected by a degree to which keywords are indigenous to the text. Table 6 provides a sense of what they look like.^15^ The indigeneity varies from one dataset to another. We want to know if or how it impacts keyword extraction.

**Table 6:** ‘Native’ versus ‘foreign’ keywords in a PubMed article. ‘Native’ keywords are ones found in the text (like those marked with an underscore), whereas ‘foreign’ keywords are those that are not. In the **keywords**  section, we find keywords supplied by humans for the abstract.

**abstract**	The notion of concordance is central to many multiple criteria techniques relying on ordinal information, e.g., outranking methods. It leads to compare alternatives by pairs on the basis of a comparison of coalitions of attributes in terms of importance. This paper proposes a characterization of the binary relations that can be obtained using such comparisons within a general framework for conjoint measurement that allows for intransitive preferences. We show that such relations are mainly characterized by the very rough differentiation of preference differences that they induce on each attribute.
**keywords**	‘concordance’, ‘outranking methods’, ‘conjoint measurement’, ‘multiple criteria analysis’, ‘nontransitive preferences’
**native**	‘concordance’, ‘outranking methods’, ‘conjoint measurement’
**foreign**	‘multiple criteria analysis’, ‘nontransitive preferences’

A success of an extractive approach depends on how many of the target keywords come from inside, because if most of them are from outside, there is no way for it to be able to find them. We highlight the issue by introducing three measures: IDP (ratio of in-document keywords), ODP (ratio of out-of-document keywords), and RIO (ratio of IDP over ODP)

$$\begin{aligned} \text {IDP}= & {} \dfrac{\# \text { of keywords found in document}}{\#\text { of keywords humans assigned to document}}\\ \text {ODP}= & {} \dfrac{\# \text {  of keywords not found in document}}{\#\text {  of keywords humans assigned to document}}\\ \text {RIO}= & {} \text {IDP}/\text {ODP}. \end{aligned}$$

RIO indicates the extent to which a given corpus depends on keywords of an internal origin: the greater the value, the more likely a keyword is found within the text. We focus on how RIO interacts with systems that employ extraction as a primary means to acquire keywords.

### Datasets

Part of the data came from the Guardian, the New York Times, PubMed Central, Reuters, Amazon, and [38]. The Guardian contained 40,000 online stories from January to late September 2014. The New York Times (NYT), approximately the size of Guardian, contained stories from January to December 2011. PubMed Central was another corpus based on abstracts in various domains found in the PubMed Central Open Access repository.^16^Reuters was a news corpus containing online articles that appeared on Reuters’ website from 2011 to 2015. The Meng dataset came from Meng et al. [38], which was made up of papers in computer science. Amazon was part of what is generally known as ‘Amazon-12K,’ a large corpus of product descriptions, each of which comes with categories or tags. In contrast to much of the previous work, which was based on documents numbered in the hundreds to thousands, we work here with considerably larger and more diverse datasets.

**Table 7:** Datasets. train (and test) = the number of documents; d.len = the average length of documents in words; keys = the average number of keywords per document; k.len = the average length of keywords in words. sup indicates datasets that contain a training block. unsup indicates those that do not (used only for unsupervised systems).

	dataset	train	test	d.len	keys	k.len	idp	odp	rio
sup	Amazon	30,000	10,000	204.25	7.36	1.83	0.22	0.78	0.28
	Guardian	20,000	10,000	791.15	7.37	1.72	0.44	0.56	0.79
	Meng	530,809	20,000	147.75	5.37	1.93	0.51	0.49	1.04
	NYT	29,986	10,000	750.85	4.41	2.46	0.50	0.50	1.00
	PubMed Central	30,000	10,000	221.90	5.26	1.80	0.60	0.40	1.50
	Reuters	14,956	10,000	672.00	10.56	1.72	0.70	0.30	2.33
unsup	kdd	–	755	74.08	5.19	1.75	0.47	0.53	0.89
	Nguyen2007	–	209	5121.67	12.09	2.10	0.82	0.18	4.51
	Schutz2008	–	1231	3550.98	46.25	1.50	0.87	0.13	6.45
	fao780	–	779	4863.16	7.98	1.62	0.59	0.41	1.43
	SemEval2010	-	243	8032.55	15.58	2.16	0.88	0.12	7.04
	PubMed	–	2000	4429.41	14.24	1.90	0.32	0.68	0.48
	Inspec	–	2000	124.36	14.11	2.22	0.59	0.41	1.44
	500N-KPCrowd	–	500	393.91	49.23	1.39	0.84	0.16	5.19
	Krapivin2009	–	2304	7855.62	5.36	2.05	0.81	0.19	4.22
	wiki20	–	20	6018.35	35.50	1.96	0.46	0.54	0.84
	fao30	–	30	4792.70	32.23	1.62	0.57	0.43	1.31
	SemEval2017	–	493	168.92	17.30	2.89	0.98	0.02	45.11
	citeulike180	–	183	4597.80	17.42	1.26	0.64	0.36	1.81
	www	–	1330	82.04	5.19	1.83	0.38	0.62	0.61
	theses100	–	100	4677.57	6.67	1.96	0.45	0.55	0.81

In addition, we made use of some 15 publicly available datasets, including 500N-KPCrowd [34], citeulike180 [37], Nguyen2007 [42].^17^ Table 7 provides a statistical profile of each of the corpora we used for this study.^18^

### Methods

In addition to TextRank (TEXTR) (Sect. 3.1), KP-miner (KPM) (Sect. 3.6), YAKE (Sect. 3.6), TopicalPageRank (TPR) (Sect. 3.7), and RAKE (Sect. 3.6), we also conducted tests for TFIDF, MAUI and ONMT-k. MAUI and ONMT-k were supervised systems.

A particular version of TFIDF^19^ we used here extracts from a document, n-grams with the length up to *n*, which do not contain punctuations, and scores them based on the TFIDF metric: TF multiplied by IDF, where TF is the term frequency and IDF is defined as $\log ({\it n}/{\it df})$, with *df* representing the document frequency, the number of documents in which a term appears, and *n*, the number of documents. TFIDF favors words that occur frequently in a small number of documents.

MAUI [37] goes through two phases to acquire keywords: candidate acquisition and ranking. In the acquisition phase, it focuses on collecting and normalizing n-grams of up to a given length. It has an option to use a controlled vocabulary. If enabled, it will work with entries in a pre-defined vocabulary in place of words found in a document. In the ranking phase, it activates features related to the text statistics, such as TFIDF, how much of the text a word covers, the location, and *keywordness,* to determine how good each candidate is. MAUI is trained with bagged decision trees [8].^20^

ONMT-k [67] is a deep learning algorithm equipped to create keywords not just from words within the document but also words from a general vocabulary found in the training data. It has the ability to generate a novel phrase which neither appeared in documents nor in gold standard labels.^21^

**Table 8:** Candidate acquisition and weighting. ‘Contiguity’ indicates whether or not a model requires candidates to be contiguous. $\text {deg}({\it w})$ = the number of times *w* occurs with other words in document *d*. $\text {freq}({\it w})$ = the frequency of *w* in *d. *

model	contiguity	candidate acquisition	candidate weighting
RAKE	+	Anything between stop words	$\text {deg}({\it w})/\text {freq}({\it w})$
YAKE	+	n-grams	Eq. 26
TFIDF	+	n-grams	TFIDF
KPM	+	anything between punctuations and stop words with a minimal frequency of 3	TFIDF$\times$ boost $\times$ position
TEXTR	+	n-grams with no limit on the length	Eq. 18
TPR	+	n-grams with no limit on the length	Eq. 30
MAUI	±	up to 3-grams, can use controlled vocabulary.	Decision Tree
ONMT-k	–	variable length	Eq. 31

Table 8 summarizes major differences and similarities among the approaches discussed above.

### Results

Table 9 shows results in F1@*k* averaged over test documents. F1@*k* represents an F1 score determined on the basis of top *k* candidates the system returned [32]. In this experiment, we set *k* to 5. Regardless of how many candidates were returned, we assumed that *k* candidates were always available: if we got less than 5, say, 3, we pretended that there were 5, with two of them being empty or zero-length keywords. We called a prediction correct only if it exactly matched one of the associated answers. Word stemming was not performed apart from MAUI. All the tokens in the corpora were uncased.

**Table 9:** Performance in F1@5

class	model	Amazon	Guardian	Meng	NYT	PubMed C.	Reuters
UNSUP	RAKE	0.0008	0.0006	0.0202	0.0022	0.0067	0.0005
	YAKE	0.0131	0.0747	0.0642	0.0848	0.0884	0.0462
	TFIDF	0.0174	0.0510	0.0715	0.0353	0.0312	0.0290
	KPM	0.0113	0.0636	0.0429	0.0443	0.0803	0.0449
	TEXR	0.0083	0.0146	0.0570	0.0173	0.0452	0.0060
	TPR	0.0053	0.0067	0.0684	0.0106	0.0610	0.0072
SUP	MAUI	0.0330	0.2060	0.1034	0.1360	0.1771	0.1306
	ONMT-k	0.3011	0.3661	0.1424	0.3012	0.1829	0.3406
RIO		0.28	0.79	1.04	1.00	1.50	2.33

class	model	kdd	Nguyen	Schutz	fao780	SemEval10	PubMed
UNSUP	RAKE	0.0385	0.0000	0.0000	0.0000	0.0000	0.0000
	YAKE	0.0822	0.1129	0.1012	0.1180	0.0776	0.0400
	TFIDF	0.0861	0.0794	0.1003	0.0856	0.0480	0.0375
	KPM	0.0477	0.1319	0.0188	0.1135	0.0674	0.0494
	TEXR	0.0345	0.0081	0.0030	0.0012	0.0018	0.0009
	TPR	0.0705	0.0146	0.0068	0.0027	0.0081	0.0020
RIO		0.89	4.51	6.45	1.43	7.04	0.48

class	model	Inspec	theses	Krapivin	wiki20	SemEval17	fao30
UNSUP	RAKE	0.0975	0.0000	0.0000	0.0000	0.0727	0.0000
	YAKE	0.0952	0.0976	0.0790	0.0642	0.0836	0.0772
	TFIDF	0.0821	0.0591	0.0594	0.0488	0.0967	0.0601
	KPM	0.0444	0.0860	0.1148	0.0574	0.0503	0.0600
	TEXR	0.0919	0.0027	0.0023	0.0000	0.0872	0.0000
	TPR	0.1738	0.0026	0.0080	0.0000	0.1660	0.0022
RIO		1.44	0.81	4.22	0.84	45.11	1.31

class	model	citeulike	www	KPCrowd
UNSUP	RAKE	0.0000	0.0401	0.0179
	YAKE	0.1427	0.0876	0.0704
	TFIDF	0.1052	0.0927	0.1024
	KPM	0.1388	0.0498	0.0687
	TEXR	0.0030	0.0477	0.0279
	TPR	0.0037	0.0758	0.0418
RIO		1.81	0.61	5.19

**Fig. 9:**
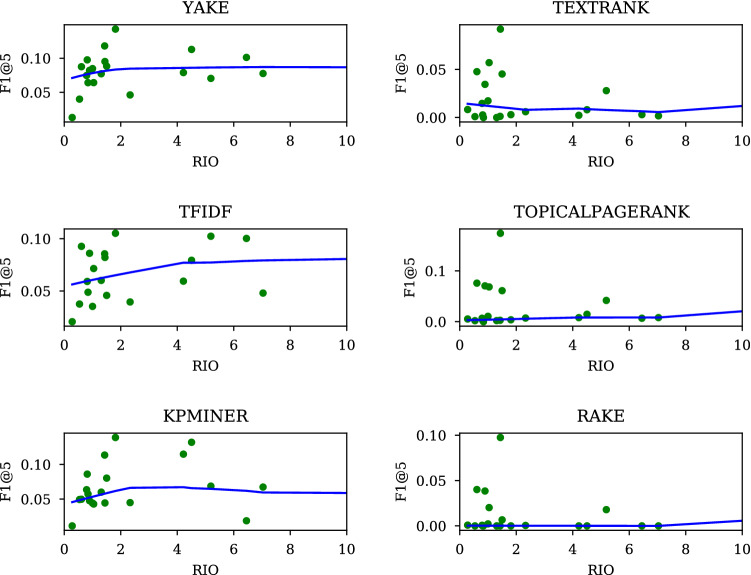
RIO vs. F1 in unsupervised systems

Found under UNSUP in Table 9 are a set of extractive systems which do not rely on supervision, and under SUP are ones that require it. For UNSUPs, F1 figures are based on their performance on the test sets, while those for SUPs are based on their performance on the same test sets after being trained on the training data.

**Fig. 10:**
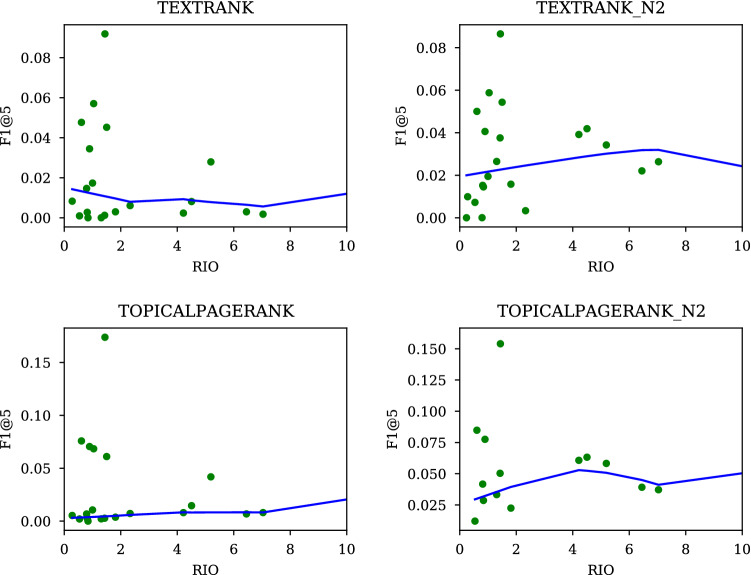
Impact of a shortening of keywords on TextRank and TopicalPageRank

**Fig. 11:**
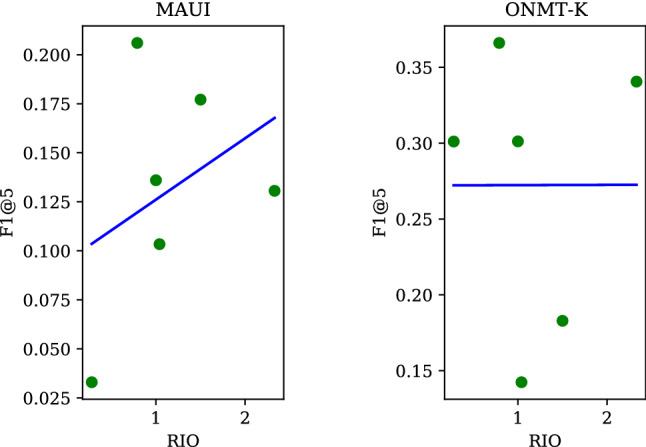
F1 vs. RIO in supervised systems

Figure 9 shows a relationship between RIO and performance. The *x*-axis represents RIO and the *y*-axis F1@5. A solid line in each panel denotes a regression line indicating how performance is affected by a change in RIO. One interesting pattern that we see in the figure is that systems on the left exhibit a behavior that consistently diverges from those on the right: the left group improves with RIO, but those on the right are not as responsive, with their performance showing no sign of improvement as RIO increases.

Table 10 shows how many words the keywords returned by a given method contained on average. RAKE has as many as 9, followed by TEXTR and TPR whose outputs are on average 3.4 words long, all of which as we observed earlier, deviated from the left group in Fig. 9, whose keywords averaged around 1 to 2 in length. The divergence in performance is most likely caused by the difference in length of keywords that they returned.

One simple way to see that this is the case is to look at what happens when TEXTR and TPR are forced to keep keywords less than 2 word long.^22^

The results are shown in Fig. 10. TEXR_N2 and TPR_N2 are tweaked versions of respective methods (whose keywords averaged around 1.9) (Table 10). This arrangement led to a visible improvement as seen in the figure, confirming that it is the average length of keywords that separates TEXTR and TPR from YAKE, TFIDF, and KPM.

**Table 10:** Average lengths of keywords

RAKE	YAKE	TFIDF	KPM	TEXTR	TPR	TEXTR_N2	TPR_N2	MAUI	ONMT-k
9.0	1.5	1.2	1.2	3.4	3.4	1.9	1.9	1.4	2.0

Finally, we move to a question of whether RIO impacts supervised systems (SUPs) as well. The result is shown in Fig. 11. The effect is more pronounced in MAUI than in ONMT-k. This is something we would have expected because of the way MAUI identifies its candidates: it looks for n-grams of up to 3 words in length, just like TFIDF and KPM. ONMT-k is largely unresponsive to RIO, which again comes as no surprise, because it ‘generates’ rather than extracts keywords from the source document. It does not care how many of the keywords originate in a source document. It is interesting that keywords ONMT-k generates are generally two word long (Table 10), indicating that the neural model implicitly learned how long they should be.

In this section, we broadly reviewed ideas that emerged over the years, with a reference to RIO. One important takeaway is that setting the length at around 2 is a critical part of making an UNSUP predictor a success. We showed that cutting the length of keywords from 3.4 to 2 improved performance of TextRank (TEXTR) and TopicalTextRank (TPR) (Fig. 10 and Table 10).^23^ Now, we know why RAKE will not and should not work as well as YAKE: keywords the former looks for average around 9, while those by the latter about 1.2–1.5.

## Conclusion

In this work, we surveyed major ideas in keyword extraction that emerged over the last 50 years, from the early 1970s, when the field was mainly led by information retrieval, to the present day which sees an escalating dominance by deep learning. The experiment has brought to light strengths and weaknesses of the methods. The fact that TFIDF and KPM ranked higher among UNSUPs suggests that a weighting scheme based on some form of TFIDF is effective, which in turn vindicates Justeson and Katz [26], who argued that there were some specific conditions for terms to qualify as an indexing term. In addition, we saw that Justeson and Katz [26]’s prediction about the length of a term: that important terms are generally two-word long, holds true across a wide range of datasets from science to business to media to bureaucracy, as well as for the corpora in Table 7 [23].^24^ The evidence is so strong that some may consider giving it a status of ‘universal constant.’ We found through RIO that some of the underperforming approaches can be fixed by forcing them to shorten keywords they generate. Setting keywords at the right length is as important as other design choices such as a weighting scheme, an observation whose significance has been underappreciated in the past literature.

Taken together, this should point to what an ideal approach in the unsupervised regime should look like: it would seek n-grams that are at most two word long, and determine their importance according to a weighting scheme more or less like TFIDF, possibly together with linguistically and statistically motivated schemes like those employed by KPM. If one wants to go beyond that, it would be wise to move to the generative regime, as it offers a capability to build keywords from within as well as from outside.

In their 2010 paper, Hasan and Ng [23] puzzled over an unexpected failure of TextRank, the state of the art at the time, to perform on par with TFIDF.^25^ The results we saw from the previous section are consistent with their findings, suggesting that their failure was most likely caused by *overly long* keywords that it produced.

An interesting area of research that has yet to be explored is an exploration of conditions under which unsupervised methods work most effectively. Granted that they lag miles behind supervised systems in terms of accuracy, that would not diminish their value: they run faster, require less resources, and are easier to deploy and adapt to novel domains. We hope to see increased research activities in this important subfield in coming years.

## Footnotes

1 Apart from their perfunctory treatment of topics that drove the field up until the early 2010s, much of their contribution centers around a re-classification of metrics long established in the community such as TFIDF and word co-occurrence, following a somewhat vague style guideline set forth by UNESCO [59] whose scientific value may be open to debate (see Firoozeh et al. [19], Fig. 4).

2 UNESCO [59] states that (1) keywords people use for a document should collectively cover major points of its content and (2) be distinctive enough to set it apart from other documents, which Firoozeh et al. [19] extended with a few more axioms, including *minimality*, *impartiality*, and *representability*, none of which concerned the length of a keyword. Their recommendation comes down to: ‘a keyword needs to be non-redundant, free of bias, descriptive, unambiguous, and in line with a communal practice.’

3 As a side note, there is another version of the approach recently explored by You et al. [66], which finds candidates via a two-stage process: locate frequent words in the document, and add neighboring words until we hit either a phrase boundary or a word which has the frequency below some threshold.

4 Wan et al. [63] are an earlier attempt to explore the utility of an affinity (adjacency) matrix to keyword extraction, where the importance of word *w* is determined by how many words there are in a text that are similar to *w*, and how many sentences the text contains that are similar to ones that include *w*.

5 SingleRank is identical to TextRank except for some small differences in the way they acquire keyword candidates. ExpandRank is another derivative of TextRank, which builds up a word graph from multiple documents (in contrast to the latter which works with a single document).

6 https://github.com/LIAAD/KeywordExtractor-Datasets.git.

7 The idea of normalizing terms with Wikipedia can be extended beyond a word sense disambiguation to socially induced associations such as *weapon* $\rightarrow$ *terrorism*, *covid-19* $\rightarrow$ *lockdown*, and *gas emission* $\rightarrow$ *global warming*.

8 A keyword is not to be confused with a summary or an abstract which is essentially an abridgment of the associated document. Any discussion on topics in automatic text summarization such as ROUGE (Lin, 2004), therefore, is of little relevance here.

9 Except for their focus on scientific articles, the difference between Yao et al. [64] and Zhang et al. [1] is mostly cosmetic from a technical point of view, and does not represent a significant departure from the previous work. A more recent effort in DL to adopt CRF can be found in Alzaidy et al. [3].

10 The way Marujo et al. [35] addressed the issue was to leverage Brown clustering (BC) [9], which enabled them to normalize corrupt spellings such as *Joooohn* *yesssss*, *Jonras of music*, or *coooooollll*, the likes of which abound in tweets. Brown clustering is a form of clustering that builds a clustering bottom–up, merging at each step clusters in a way that would maximize mutual information (MI). To normalize word *Joooohn* involves finding a cluster that when combined with the word, gives a maximum MI. BC has an important side effect of reducing vocabulary. Marujo et al. [35] found that MAUI, when trained on a BC-induced representation, saw an improvement by 25 percentage points over the default model (which does not apply normalization).

11 We mean by ‘extractive’ a class of approaches that aim at spotting interesting words within a text, and by ‘classificatory’ those that find keywords by classifying the text.

12 It represents quite a departure from Meng et al. [38] which cannot handle more than one keyword at a time.

13 https://github.com/boudinfl/pke.git.

14 https://radimrehurek.com/gensim/models/ldamodel.html.

15 Other than what we have in Table 6, *native* keywords include technical and indexing terms (Sect. 2.1) and *foreign *keywords, annotation tags used in the news media (‘business,’ ‘politics,’ ‘world’), and categories in Wikipedia (‘Living People,’ ‘Nobel Peace Prize Laureates’).

16 We bulk-downloaded data from ftp://ftp.ncbi.nlm.nih.gov/pub/pmc/oa_bulk/. We retained only keywords, abstracts, and DOI descriptors.

17 https://github.com/LIAAD/KeywordExtractor-Datasets.git.

18 There is a peculiar divide among the datasets, one between SUPs and UNSUPs. The former tend to have RIOs significantly lower than the latter. Some may argue that the former have in-text and out-of-text keywords more in balance in comparison to the latter, which are skewed towards in-text keywords. This may get some people to question the validity of the datasets, wondering whether the latter datasets are *natural.* In this connection, Turney [57] makes an interesting observation. He reported that in-text keywords accounted for 73% in Journal of the International Academy of Hospital Research, 74.9% in Psycoloquy, 91.7% in The Neuroscientist, 78.8% in The Journal of Computer-Aided Molecular Design, and 87.4% in Behavioral & Brain Sciences Preprint Archive. The corresponding RIOs are: 2.70 (Hospital Research), 3.0 (Psycoloquy), 11.5 (Neuroscientist), 3.76 (Molecular Design), and 6.64 (Brain Sciences), which show that ‘naturally occurring’ datasets can also have high RIOs.

19 https://github.com/boudinfl/pke.git.

20 Turney [57, 58] introduced an idea similar to MAUI (called GenEX, based on a genetic algorithm), 5 to 7 years prior to the latter’s appearance. It used textual cues such as the location of a keyword (whether it appeared early or late in the text), and its in-text frequency. GenEX returns a set of values which are used to score a candidate.

21 ONMT-k is a derivative of Gu et al. [22], who proposed an idea of ‘copying mechanism,’ where the decoder is allowed to peek into the input to decide whether to reuse part of it as it generates an output. An innovation of ONMT-k lies with the simple idea of training the model on a concatenation of ground truth keywords as a target, enabling the model to cope with keywords which come in varying numbers. ONMT-k measures the quality of an output (a sequence of keywords $k_1,\dots ,k_n$) by

$$\begin{aligned} P(k_1,\dots ,k_n \mid x, \theta ) = \sum _{{\it w} \in k_1,\dots ,k_n} \log p_f( {\it w} \mid x, \theta ).\quad \quad \quad \quad \quad \quad \quad \quad (31); \end{aligned}$$

*w* is a word token. $p_f({\it w} \mid x, \theta )$ is the probability of emitting *w* under model $p_f$ (a neural network representing ONMT-k) with parameters $\theta$.

22 RAKE was excluded here as there was no way to tweak it to accommodate the length restriction.

23 Another possible use scenario of RIO would be to use it as a device to estimate how likely one would succeed with an unsupervised method (UNSUP) on a given dataset. If the value is low, one may be better off not to use it. From what we see in Figs. 9 and 10, it would not be a stretch to say that the dividing line lies somewhere around 1 (i.e., IDP and ODP are in balance): if RIO is above 1, there will be some chance that an UNSUP may work; otherwise, the use of SUPs is advised.

24 We found that the average length of keywords in 20 most commonly used datasets (https://github.com/LIAAD/KeywordExtractor-Datasets.git) was at 1.82, fairly close to what we found in Table 7.

25 See *ibid.,* Table 2 and Fig. 1.
